# Genetic characterization of gut-associated *Clostridium perfringens* isolates from Austrian animals using a combination of established and novel methods

**DOI:** 10.3389/fmicb.2026.1944327

**Published:** 2026-09-25

**Authors:** Michael Blümlinger, Lukas Schwarz, Joachim Spergser, Adriana Cabal-Rosel, Werner Ruppitsch, Maximilian Collatz, Sascha D. Braun, Stefan Monecke, Ralf Ehricht, Andrea Buzanich-Ladinig, Igor Loncaric

**Affiliations:** 1 Unit Swine Medicine, Clinical Centre for Population Medicine in Fish, Pig and Poultry, Clinical Department for Farm Animals and Food System Science, University of Veterinary Medicine Vienna, Vienna, Austria; 2 Unit Microbiology, Centre of Pathobiology, Department of Biological Sciences and Pathobiology, University of Veterinary Medicine Vienna, Vienna, Austria; 3 Division Public Health, Austrian Agency for Health and Food Safety, Institute for Surveillance and Infectious Disease Epidemiology, Vienna, Austria; 4 Institute of Hygiene and Medical Microbiology, Medical University Innsbruck, Innsbruck, Austria; 5 Faculty of Food Technology, Food Safety and Ecology, University of Donja Gorica, Podgorica, Montenegro; 6 RNA Bioinformatics and High-Throughput Analysis, Friedrich Schiller University Jena, Jena, Germany; 7 Bioinformatics Core Facility Jena, Friedrich Schiller University Jena, Jena, Germany; 8 Leibniz-Institute of Photonic Technology (Leibniz-IPHT), Member of the Leibniz Center for Photonics in Infection Research (LPI), Jena, Germany; 9 InfectoGnostics Research Campus, Jena, Germany; 10 Center for Translational Medicine (CETRAMED), Jena University Hospital, Friedrich Schiller University Jena, Jena, Germany; 11 Institute of Physical Chemistry, Friedrich Schiller University Jena, Jena, Germany

**Keywords:** antimicrobial resistance, *Clostridium perfringens*, DNA microarray, maximum likelihood tree, molecular genetic characterization, multi locus sequence typing, virulence associated toxins, whole genome sequencing

## Abstract

**Introduction:**

*Clostridium perfringens* (*C. perfringens*) is a pathobiont of the intestinal microbiota that can cause severe systemic and enteric diseases. *C. perfringens* colonizes a variety of hosts and environmental niches, which is the theorized reason for its large genome, low guanine-cytosine content, and small stable core genome. Over 20 putative virulence factors have been described, but only six are included in the used typing scheme. Epidemiological analysis is limited, and to the authors’ knowledge, this is the first study for Austria. Furthermore, *C. perfringens* frequently harbors different and/or multiple genes associated with antibiotic resistance (AMR genes) targeting various antibiotic classes.

**Methods:**

A novel DNA microarray – targeting 15 *C. perfringens*-associated genes – was evaluated against a published multiplex PCR (*n* = 143) and *in silico* analysis of Whole Genome Sequences (WGS) (*n* = 20). Multi Locus Sequence Typing (MLST) was performed (*n* = 124), the results were analyzed using the goeBURST algorithm, and a maximum likelihood tree was constructed. Genomes of this study and publicly available (*n* = 44) were analyzed *in silico* for AMR genes.

**Results:**

The DNA microarray detected all target genes except *becA* and *becB*, which were absent across all tested strains and methods. The most frequent virulence factors were *cpa* (*n* = 143), *tpi* (*n* = 132), and *cpb2* (*n* = 104). MLST revealed 77 Sequence Types (STs) – including 42 novel STs – clustered into six Clonal Complexes (CCs). CC39 was the most abundant and exclusively associated with pork/swine. At least one AMR gene was present in 58.7% of genomes and multiple AMR genes in 34.8%. AMR genes associated with resistance to tetracycline, aminoglycosides, pleuromutilin, lincosamides, macrolides, and streptogramins but non to beta-lactams were detected. Notably, one isolate carried *lsa*(E), which was not previously described for *C. perfringens*.

**Conclusion:**

The DNA microarray showed detection rates comparable to those of multiplex PCR and WGS analysis while being less labor-intensive and more cost-effective. This is the first epidemiological study on *C. perfringens* for Austria. We described 42 novel STs and six CCs of which CC39 appears to be porcine-associated. The *lsa*(E) gene was detected in a *C. perfringens* isolate.

## 1 Introduction

*Clostridium perfringens* (*C. perfringens*), a strictly anaerobic, rod shaped, Gram positive and endospore forming bacterium, is commonly associated with a variety of human and animal diseases (Kiu and Hall, 2018; McClane et al., 2025). These include serious systemic conditions such as gas gangrene and enteric diseases, including, food poisoning, non-food born diarrhea and necrotic enteritis/ enterocolitis (Nagahama et al., 2019; Shrestha et al., 2019). *C. perfringens* can also cause antibiotic-associated diarrhea, which, however, in humans is more commonly associated with *Clostridioides difficile* (Asha et al., 2006).

*C. perfringens* has a genome size ranging from 2.9–4.1 mega bases and an average guanine-cytosine content of 27.8–28.6% (Moore and Lacey, 2019). *C. perfringens* pathogenicity largely depends on the production of virulence factors, such as toxins and extracellular enzymes. To date, over 20 different putative virulence factors have been identified, but the exact function and relevance of them is not yet completely revealed (Revitt-Mills et al., 2019; Camargo et al., 2024). *C. perfringens* can be typed into seven distinct types based on the presence, absence and combinations of six defined toxin genes. The typing scheme is not definite and it was last updated in 2018, when two novel toxin gene types were added (Rood et al., 2018). Evidence is mounting that the pore-forming toxin encoded by *netF* can play a role in canine acute hemorrhagic diarrhea syndrome and foal necrotizing enteritis (Mehdizadeh Gohari et al., 2020). A recent study reported a detection rate of 79.0% in *C. perfringens* isolated of pig fecal samples (Zekun et al., 2026). Besides a wide distribution of *C. perfringens* in the environment (e.g., soil, decomposing organic matter and sewerage), the bacterium can be part of the physiological microbiota of healthy humans and animals. Therefore, the mere isolation of *C. perfringens* from clinical specimens of diarrhetic patients is not sufficient to unequivocally attribute the clinical disease to this pathogen (Mehdizadeh Gohari et al., 2021); further diagnostic work-up is consequently recommended. In routine diagnostics, toxin genotyping according to Rood et al. (2018) *via* single or multiplex PCR is performed, sometimes including additionally *cpb2*. To gather more knowledge on the relevance of other virulence factors not included in the used typing scheme, a larger panel is needed for routine diagnostics. Numerous toxin genes – except *plc/ cpa* and sometimes *cpe –* are located on conjugative plasmids (Cornillot et al., 1995; Li et al., 2013). The loss and gain of toxin genes *via* plasmid exchange is described for *C. perfringens* (Li et al., 2013). Furthermore, toxin genes are sometimes encoded on plasmids closely related to the paradigm tetracycline resistance plasmid pCW3. Other mobile antimicrobial resistance elements are encoded on plasmids as well (e.g., chloramphenicol resistance integrative mobilizable elements, bacitracin resistance integrative conjugative element and lincomycin resistance transferable insertion sequence) (Adams et al., 2018). For *C. perfringens*, a plethora of resistance genes are described. The main resistance mechanisms include chemical modifications, generic and specific efflux pumps, target site methylation, mutational modification and target site protection. Detection rates of antimicrobial resistance genes can vary depending on geographic distribution and local practices in antimicrobial substance usage (Schwarz et al., 2018).

*C. perfringens* has a highly variable and open pangenome, as only 12.6% of the genome is present in most of the strains and therefore regarded as core genome. The rather small stable parts of the genome may reflect the ability of *C. perfringens* to inhabit vastly different environments and hosts, which leads to active interaction with changing microbiomes with potential high frequency of gene loss or gain events. Supporting this claim is the high number of genetic elements associated with horizontal gene transfers (i.e., transposases, integrases and prophages) present in the genome of *C. perfringens* (Kiu et al., 2017). Various phenotypic and genotypic typing techniques for *C. perfringens* have been published in order to examine the relatedness and epidemiology of this bacterium (Abdel-Glil, 2020). Multi Locus Sequence Typing (MLST) is an easily accessible and relatively inexpensive method that relies on comparing distinct housekeeping genes. Currently there are at least four different MLST schemes published for *C. perfringens* (Jost et al., 2006; Neumann and Rehberger, 2009; Hibberd et al., 2011; Xiao et al., 2012), whereas the public database PubMLST adopted the eight gene MLST by Xiao et al. (2012) (Jolley et al., 2018). Due to the existence of multiple MLST schemes, it is difficult to compare results from publications that use different schemes. Nevertheless, there are approximately 1,700 isolate entries on PubMLST^1^, out of which approximately 310 originate from Europe. At the start of this study, no data on the epidemiology of Austrian *C. perfringens* strains was available. Therefore, one of the study’s goals was to examine the relationship between the MLST analysis of *C. perfringens* strains isolated from the feces of various animal species. However, data on the occurrence and distribution of antimicrobial resistance associated (AMR) genes in Austrian *C. perfringens* strains remain limited. Therefore, Whole Genome Sequences (WGS) of selected isolates were examined *in silico* for the presence of AMR genes. Since more than 20 virulence factors have been described for *C. perfringens* (Revitt-Mills et al., 2015; Camargo et al., 2024) and only six toxin genes are regularly analyzed in routine diagnostics (Rood et al., 2018), we established a DNA microarray platform for simultaneous detection of multiple genes within a single reaction for *C. perfringens*. In addition, a methodological gap was identified between the molecular testing required for toxins, which uses mono- or multiplex PCR, and Next Generation Sequencing (NGS). DNA microarray based assays can be performed in parallel at scale, are faster and less expensive than NGS, and are more practical than combining numerous multiplex PCRs to obtain comparable information.

## 2 Materials and methods

### 2.1 *Clostridium perfringens* isolates, cultivation and DNA extraction

In total, 136 non repetitive clinical *C. perfringens* isolates (porcine, *n* = 89; canine, *n* = 10; bovine, *n* = 10; avian, *n* = 10; ovine, *n* = 9; caprine, *n* = 5; lama, *n* = 1; and not recorded, *n* = 2) and 7 reference strains were used in the presented study (Supplementary Table 1). All clinical isolates were gut-associated (i.e., fecal samples, rectal swabs or intestinal loop content), originated from Austrian animals and were isolated during routine diagnostics at the Microbiology Unit, University of Veterinary Medicine Vienna, Austria. Therefore, the samples were not subject to the reporting obligations of the ethics and animal welfare commission of the University of Veterinary Medicine Vienna. Strains were preserved in cryoconservative medium at −70 °C until further use.

All isolates were recultivated overnight on Colombia III Blood Agar supplemented with 5% Sheep blood (Becton Dickinson Austria GmbH, Vienna, Austria) under anaerobic conditions (GasPak EZ Anaerobe Container System Sachets, Becton Dickinson Austria GmbH, Vienna, Austria) at 37 °C. Colonies were confirmed as *C. perfringens* by matrix assisted laser desorption ionization-time-of-flight (MALDI ToF) mass spectrometry (MS) (Microflex LT, Bruker Corporation, Billerica, United States). Single colonies were picked, re-streaked on the same medium and incubated overnight under previously described conditions.

Approximately one inoculation loop (Inoculation loops 1 μL, Sarstedt, Nürmbrecht, Germany) of biomaterial of the clonal overnight cultures was used for DNA extraction. Therefore, biomaterial was directly scraped off the agar plate. Afterwards, the cells got suspended in an enzymatic lysis buffer for Gram positive bacteria (Reagent 1–1, INTER-ARRAY by fzmb GmbH, Bad Langensalza, Germany) for 30–60 min at 37 °C and 550 rounds per minute (rpm) (Thermo Shaker Sc-24, Kisker Biotech GmbH & Co. KG, Steinfurt, Germany). Afterwards 200 μL enzymatic lysis buffer (Buffer AL, QIAGEN GmbH, Hilden, Germany) supplemented with 35 μL proteinase K (Proteinase K Solution, QIAGEN GmbH, Hilden, Germany) was added and incubated for a minimum of 60 min or until the suspension cleared at 56 °C and 550 rpm. Next, DNA was extracted with a commercial kit (DNeasy Blood and Tissue Kit, QIAGEN GmbH, Hilden, Germany) according to manufacturer’s instructions. To increase the DNA quantity the eluate was centrifuged for 30 min in a vacuum centrifuge (GeneVac miVac Duo concentrator, Fisher Scientific (Austria) GmbH, Vienna, Austria) with default settings for complete alcohol evaporation. All samples were checked for DNA quantity and quality *via* a spectrophotometer (NanoDrop 2000 Spectrophotometer, Fisher Scientific (Austria) GmbH, Vienna, Austria) according to the manufacturer’s instructions (Supplementary Table 2).

### 2.2 Whole genome sequencing

Twenty *C. perfringens* isolates were selected for whole-genome sequencing (WGS) based on DNA Microarray results. Strains displaying various virulence-associated genes were selected for further characterization by WGS. Whole Genome Sequencing was done on ready-to-sequence libraries prepared by Nextera XT DNA Library Preparation Kit (Illumina, Inc., San Diego, California, United States). Paired-end sequencing was performed on the Illumina Nexseq2000 (Illumina, Inc., San Diego, California, United States) platform, with a read length of 2 × 150 bp and 30X as target coverage. Reads were trimmed and assemblies generated using SPAdes 3.15.5. (Prjibelski et al., 2020) with default settings. Contigs were filtered for a minimum coverage of 5 × and a minimum length of 200 bp using Ridom SeqSphere+ software v.9.0.3 (Jünemann et al., 2013).

Furthermore, 24 publicly available *C. perfringens* genomes (human, *n* = 9, including one from a mummy; wild avian, *n* = 3; ovine, *n* = 2; porcine, *n* = 2; canine, *n* = 2; equine, *n* = 2; environmental, *n* = 1; food, *n* = 1 and not recorded, *n* = 2) from the Gen Bank NCBI Genome (Sayers et al., 2025) (Supplementary Table 1) were used for additional genetic analysis and phylogenetic tree construction.

### 2.3 Molecular characterization of *Clostridium perfringens* isolates

#### 2.3.1 Multiplex toxin-gene-typing PCR

A multiplex toxin-gene typing PCR was performed on all cultured *C. perfringens* strains (Supplementary Table 1) using the standard typing scheme and a modified version of the protocol described by Rood et al. (2018). Briefly oligonucleotide primers for *netB, etx, iap, plc, cpe* and *cpb* in the concentrations 0.6 μM, 0.44 μM, 0.5 μM, 0.4 μM, 0.3 μM and 0.3 μM, respectively, were used (Table 1). The desired reaction volume was 20 μL. For half of the reaction volume (i.e., 10 μL) a buffered polymerase premix (OneTaq Quick-Load 2X Master Mix with Standard Buffer, New England BioLabs GmbH, Frankfurt am Main, Germany) was added. Additionally, 0.5% of the reaction volume polymerase (OneTaq Quick-Load DNA Polymerase, New England BioLabs GmbH, Frankfurt am Main, Germany) was added. Nuclease free water was added to the desired final volume. Finally, the reaction mix was loaded with 1 μL of DNA template. PCR was performed (Mastercycler nexus gradient/ Mastercycler nexus eco, Eppendorf SE, Hamburg, Germany) with an initial denaturation step of 95 °C for 5 min, followed by 35 cycles of 95 °C for 1 min, 55 °C for 1 min and 72 °C for 1 min. For each reaction a positive control (i.e., DNA extracts of analyzed strains included in this study – NCTC8533 for *cpa, cpb* and *etx*; NCTC8084 for *cpa* and *iap/ ibp*; NCTC8798 for *cpa* and *cpe* and 18_10724 for *cpa* and *netB*) and negative control (i.e., nuclease free water) was included. Nucleic acid amplicons were resolved by gel-electrophoresis (2% Agarose; LabQ Standard Agarose LE, LabConsulting GmbH, Vienna, Austria) for 50 min at 300 Volts (PowerPac 300/ PowerPac Basic, Bio-Rad Laboratories GmbH, Feldkirchen, Germany; Owl A2, Fisher Scientific (Austria) GmbH, Vienna, Austria), stained in ethidiumbromid (500 μL of 10 mg/mL ethidium bromide - Carl Roth GmbH + Co. KG, Karlsruhe, Germany – dissolved in 2000 mL *Aqua destillata*), transilluminated by ultraviolet light and photodocumented (ChemiDoc MP Imaging System, Bio-Rad Laboratories GmbH). A size marker (1 kb Plus DNA Ladder, New England BioLabs GmbH, Frankfurt am Main, Germany) was used.

**Table 1:** Primers and probes used in this study.

Application	Gene	Primer or probe	Sequence (5′ - 3′)	Reference
Multiplex-Toxin-Gene-Typing PCR	*netB*	JRP6656	CTTCTAGTGATACCGCTTCAC	Rood et al. (2018)
		JRP6655	CGTTATATTCACTTGTTGACGAAAG	
	*etx*	JRP4234	CCACTTACTTGTCCTACTAAC	
		JRP4235	GCGGTGATATCCATCTATTC	
	*iap*	JRP5507	GGAAAAGAAAATTATAGTGATTGG	
		JRP5508	CCTGCATAACCTGGAATGGC	
	*plc/ cpa*	JRP4232	GCTAATGTTACTGCCGTTGACC	
		JRP4233	CCTCTGATACATCGTGTAAG	
	*cpe*	JRP5179	GGAGATGGTTGGATATTAGG	
		JRP5180	GGACCAGCAGTTGTAGATA	
	*cpb*	JRP5181	GCGAATATGCTGAATCATCTA	
		JRP5182	GCAGGAACATTAGTATATCTTC	
Multi Locus Sequence Typing	*gyrB*	gyrB-F	ATTGTTGATAACAGTATTGATGAAGC	Xiao et al. (2012)
		gyrB-R	ATTTCCTAATTTAGTTTTAGTTTGCC	
	*sigK*	sigK-F	CAATACTTATTAGAATTAGTTGGTAG	
		sigK-R	CTAGATACATATGATCTTGATATACC	
	*sod*	sod-F	CAAAAAAAGTCCATTAATGTATCCAG	
		sod-R	TTATCTATTGTTATAATATTCTTCAC	
	*groEL*	groEL-F	TACAAGATTTATTACCATTACTTGAG	
		groEL-R	CATTTCTTTTTCTGGAATATCTGC	
	*pgk*	pgk-F	GACTTTAACGTTCCATTAAAAGATGG	
		pgk-R	CTAATCCCATGAATCCTTCAGCGATG	
	*nadA*	nadA-F	ATTAGCACATTATTATCAAATTCCTG	
		nadA-R	TTATATGCCTTTAATCTTAAATCCTC	
	*colA*	colA-F	ATTAGAAAGTTTATGTACAATAGGTG	
		colA-R	AAGACATTCTATTATTTCTATCGTAAGC	
	*plc/ cpa*	plc-F	AGGAACTCATGATTGTAACTC	
		plc-R	GGATCATTACCCTCTGATACATCGTG	
DNA Microarray	*becA*	hp:becA-01	CCTATATTGGTTCATTTAAAGTTGCCAA	this study
	*becB*	hp:becB-01	TGAAACTTCAAATACTGTAACTAACGAAAC	
		hp:becB-02	CTGAGAAACCTTTAGAAGTCGAATTAACA	
	*plc/ cpa*	hp:cpa_01	AATAGTTGGTATTTAGCTTATTCTATACCTGA	
		hp:cpa_02	TCTTACACGATGTATCAGAGGGTAA	
	*cpb*	hp:cpb-01	TGAAAGCGAATATGCTGAATCATCTAC	
		hp:cpb-02	ACAACCCTGTATATGGAAATGAAATGT	
	*cpb2*	hp:cpb2-01	ACATAGTAACTCCTTCAATGAGAGCT	
		hp:cpb2-02	TCCTACACCAAGTTTCAGAACTCA	
		hp:cpb2-03	ACACCATCATTTAGAACTCAAGTTTGT	
		hp:cpb2-11	ACTTCAGGCTCAAGATCATTAAGTG	
		hp:cpb2-12	AGATGATGTTAAAGCTTCTCAAATGGT	
		hp:cpb2-13	TGACATTAGAGCTTCTCAATGGGT	
	*cpd*	hp:cpd-03	CACTTAAAGGATTTATTCCATCAGGAAGA	
		hp:cpd-04	TCATACTCTATAGGTGGAGGAATAGATACA	
	*cpe*	hp:cpe-01	CTAATGAAACAGGTACCTTTAGCCAA	
		hp:cpe-02	ATCCTTGATTTAGCTGCTGCTACA	
		hp:cpe-03	CGTGGTAGTATATCTGATGATAGAGCA	
		hp:cpe-04	AGAAATTCTTGATTTAGCTACTTCTACAGAA	
	*etx*	hp:etx-01	GCAACTACTACTCATACTGTGGGAA	
		hp:etx-02	ACTCATAATGTCCCTTCACAAGATATAC	
	*iap*	hp:iap-01	CTGAAGCTTCTATTGTAAACAGTCTTGA	
	*ibp*	hp:ibp-01	AGCACAGGATAATCAAATTGGTAATAACT	
		hp:ibp-02	GCACATAACAAATATTATGCAGATATAAAGC	
	*netB*	hp:netB-01	TTCTAGTGATACCGCTTCACATAAAG	
		hp:netB-06	GGACGGTTATAATATAGATTCTTATCATGCT	
	*pfoA*	hp:pfoA1-01	GTGGAAAATAGACCTACAATCTTAATGGT	
		hp:pfoA1-02	GAATGGTGGAGAGATGTTATAAGTGAA	
		hp:pfoA2-01	GGAGAACTGTTGTAAATGAGCATAATGT	
	*recA*	hp:recA-01	CAAATGGATGCTGTTTCTACTGGA	
	*tpeL*	hp:tpeL-01	TCATCTAATATGACAGCAGCTATTAGTTC	
		hp:tpeL-02	TGGGACTAAAGAAGATAATTGGATTCTT	
	*tpi*	hp:tpi-01	CTAATGAAACAATAGCTGCTATAAGAGCT	

#### 2.3.2 Multi locus sequence typing

Conventional MLST was performed on an initial cohort of 80 strains according to the protocol of Xiao et al. (2012). The remaining isolates were not subjected to MLST because of logistical constraints associated with the assay and scale of the collection. Additionally *in silico* MLST was performed on the 44 genomes either generated (*n* = 20) or publicly available (*n* = 24) (Supplementary Table 1).

For conventional MLST the genes *gyrB, sigK, sodA, groEL, pgk, nadA, colA* and *plc* (Table 1) were amplified according to the published protocol (Xiao et al., 2012). A target reaction volume of 30 μL was used, therefore 15 μL of a buffered polymerase premix (OneTaq Quick-Load 2X Master Mix with Standard Buffer, New England BioLabs GmbH, Frankfurt am Main, Germany), 10.5 μL nuclease free water, 1.5 μL forward, 1.5 μL reverse primer (Table 1; 1 μM final primer concentration) and 1.5 μL target DNA was used per reaction. PCR was performed (Mastercycler nexus gradient/ Mastercycler nexus eco, Eppendorf SE, Hamburg, Germany) with an initial denaturation step of 94 °C for 5 min, followed by 30 cycles of 94 °C for 0.5 min, 50–55 °C for 0.5 min (depending on the amplified target gene sequence; i.e. *sodA* and *plc 50 °C; gyrB, sigK, groEL, nadA* and *colA* 52 °C; *pgk* 55 °C) and 72 °C for 1 min.

Subsequently the amplicons got enzymatically purified *via* a commercial Kit (Exo-CIP Rapid PCR Cleanup Kit, New England BioLabs GmbH, Frankfurt am Main, Germany) according to the manufacturer’s instructions. Briefly, 1 μL of thermolabile exonuclease I (Exo-CIP A, New England BioLabs GmbH, Frankfurt am Main, Germany) and 1 μL of thermolabile calf intestinal phosphatase (Exo-CIP B, New England BioLabs GmbH) were added to 5 μL DNA product and were incubated with the following thermocycler (Mastercycler nexus gradient/ Mastercycler nexus eco, Eppendorf SE, Hamburg, Germany) conditions: 37 °C for 4 min and 80 °C for 1 min.

Afterwards samples were prepared for transport to an external laboratory (LGC Genomics GmbH, Berlin, Germany) for sequencing according to the laboratory’s instructions. The external laboratory provided the sequencing results of the automated chromas readings as text files in FASTA format and the generated chromas files as standard chromatogram format.

The raw read Sanger or WGS sequences were checked in single or batch on the website PubMLST^2^ (Jolley et al., 2018). Novel alleles were uploaded in the manually curated database according to the website’s instructions. If there were errors or other inconsistencies the original files were checked with a software (Chromas, Technelysium Pty Ltd., South Brisbane, Australia) and explainable errors were corrected manually (i.e., double readings of a single nucleobase peak). Otherwise, sequencing was redone.

#### 2.3.3 DNA microarray

A customized DNA microarray was produced by INTER-ARRAY by fzmb GmbH (Bad Langensalza, Germany) to detect 15 different *C. perfringens* associated genes using 33 specific probes (Table 1). All probes were present on the array at least in duplicates. In total 143 strains (Type strain, *n* = 7; porcine, *n* = 89; canine, = 10; caprine, *n* = 5; bovine, *n* = 10; ovine, *n* = 9; lama, *n* = 1; avian, *n* = 10 and not recorded, *n* = 2) were analyzed with this DNA microarray (Supplementary Table 1).

First a linear amplification was performed according to the manufacturer’s instructions to label the target DNA. Briefly, oligonucleotide primers for the 33 different probes were added in a concentration of 0.135 μM each to a mastermix. Target reaction volume was 10 μL, therefore 3.9 μL of labeling buffer (Reagent 2–0, INTER-ARRAY by fzmb GmbH), 0.1 μL of labeling enzyme (Reagent 2–2, INTER-ARRAY by fzmb GmbH), 1 μL of the mastermix and 5 μL target DNA were added into a reaction tube. Only target DNA with a concentration higher than 50 ng/μL and a purity of at least 1.5 260/280 and 1.5 260/230 according to spectrophotometer measurement were used (Supplementary Table 2). The labeling reaction was performed (Mastercycler nexus gradient/ Mastercycler nexus eco, Eppendorf SE, Hamburg, Germany) with an initial denaturation step of 95 °C for 5 min, followed by 50 cycles of 96 °C for 1 min, 50 °C for 20 s and 72 °C for 40 s.

Second the labeled DNA amplicons were hybridized onto the probes of the DNA microarray according to manufacturer’s instructions. Briefly, the array reaction tubes were prepared by firstly incubating them with 150 μL nuclease free water for 5 min at 50 °C and 550 rpm (BioShaker iQ, QINSTRUMENTS GmbH, Jena, Germany) and secondly by incubating them with 150 μL hybridization buffer (Reagent 3–1, INTER-ARRAY by fzmb GmbH) for 5 min at 50 °C and 550 rpm. Subsequently the hybridization followed by incubating 100 μL hybridization-mix (i.e., 10 μL labeled DNA amplicon + 90 μL hybridization buffer; Reagent 3–1, INTER-ARRAY by fzmb GmbH) for 60 min at 50 °C and 550 rpm. For this step the reaction tubes were sealed with well plugs to avoid evaporation of reagents. Thereafter two washing steps followed. First the reaction tubes were rinsed with 150 μL washing buffer 1 (Reagent 3–2, INTER-ARRAY by fzmb GmbH) and then they were incubated with 150 μL washing buffer 1 (Reagent 3–2, INTER-ARRAY by fzmb GmbH) for 10 min at 30 °C and 550 rpm. Afterwards detection followed by incubating 100 μL detection-mix (i.e., 99 μL conjugate buffer; Reagent 4–2, INTER-ARRAY by fzmb GmbH + 1 μL horse radish peroxidase conjungate 100x; Reagent 4–1, INTER-ARRAY by fzmb GmbH) for 15 min at 30 °C and 550 rpm. Two washing steps followed. Firstly, the reaction tubes were rinsed with 150 μL washing buffer 2 (Reagent 4–3, INTER-ARRAY by fzmb GmbH) and secondly, they were incubated with 150 μL washing buffer 2 (Reagent 4–3, INTER-ARRAY by fzmb GmbH, Bad Langensalza, Germany) for 2 min at 30 °C and 550 rpm. Finally, staining was performed by incubating 100 μL of horse radish peroxidase substrate solution blue (Reagent 5–1, INTER-ARRAY by fzmb GmbH) for 8 min at room temperature. After each step the reaction tubes were cleared of the previous reagent.

Next, the array stripes were placed onto an automated reader (INTER-ARRAY Reader, INTER-ARRAY by fzmb GmbH) to measure the pixel density of the spots where the probes are attached in comparison to the surrounding background. Pixel density results for all probes were generated by a designated software (Inter-Vision Genotyping 1.1.2, INTER-ARRAY by fzmb GmbH). The measurements yielded a value between 0 and 1, whereas a value over 0.3 was regarded as positive. Raw reads were manually curated and when all corresponding duplicate probes for a gene yielded a pixel density above 0.3 a positive result has been assigned to the corresponding gene.

### 2.4 Bioinformatic analysis

Sequences in FASTA format of annotated, trimmed and concatenated allelic MLST profiles generated in this study of 124 investigated strains were downloaded from PubMLST (Jolley et al., 2018) as plain text files. Subsequently a maximum likelihood tree was constructed with the concatenated allele sequences using the software MEGA12 (Kumar et al., 2024), with default settings, Tamura-Nei model (Tamura and Nei, 1993) and 1,000 standard bootstraps value. Phylogroup assignation was performed according to the branch affiliation to publicly available genomes, which was described by Abdel-Glil et al. (2021).

The maximum likelihood tree was modified for better readability (InkScape, Version: v.1.4.2) and further data was added manually (Microsoft Excel, Version: 2502).

Allelic profiles of the 124 strains analyzed *via* MLST were downloaded from PubMLST. GoeBurst analysis of the generated allelic profiles was performed as described by Francisco et al. (2009) with the software PHYLOViZ2 (Nascimento et al., 2017). The programs default settings were applied.

Isolates were regarded as clonal when Sequence Types (STs) did not differ or if they differed in one allele and Clonal Complexes (CCs) were attributed manually. Strains were considered as close relatives when the STs differed in two alleles or less.

All WGS generated in this study (*n* = 20) were screened for the presence of AMR genes using AMRfinderPlus (Software version: 4.0.23, Database version: 2025-07-16.1; Feldgarden et al., 2021) according to the programmers instructions without the add-in for virulence factors. Additionally, publicly available WGS (*n* = 24) were screened as well and were used for comparison (Supplementary Table 1).

In addition, all WGS used in this study were screened *in silico* for the presence of virulence associated genes and available variants using the Standard Nucleotide Basic Local Alignment Search Tool (BLAST) algorithm optimized for highly similar sequences (National Center for Biotechnology Information, 2013), with 90% identity and 30% query coverage. In total 45 virulence associated genes and gene variant sequences were used as described by Abdel-Glil et al. (2021). The genomes of WGS isolates were deposited under PRJNA1495884 in the NCBI BioProject database and under PRJEB121207 in the European nucleotide archive.

## 3 Results

### 3.1 Multiplex toxin-gene-typing PCR

In the multiplex PCR 143 strains were tested and all yielded a positive result for *plc/ cpa*, 4 for *cpb*, 6 for *etx*, 1 for *iap/ ibp*, 1 for *cpe* and 2 for *netB* (Figure 1).

**Figure 1:**
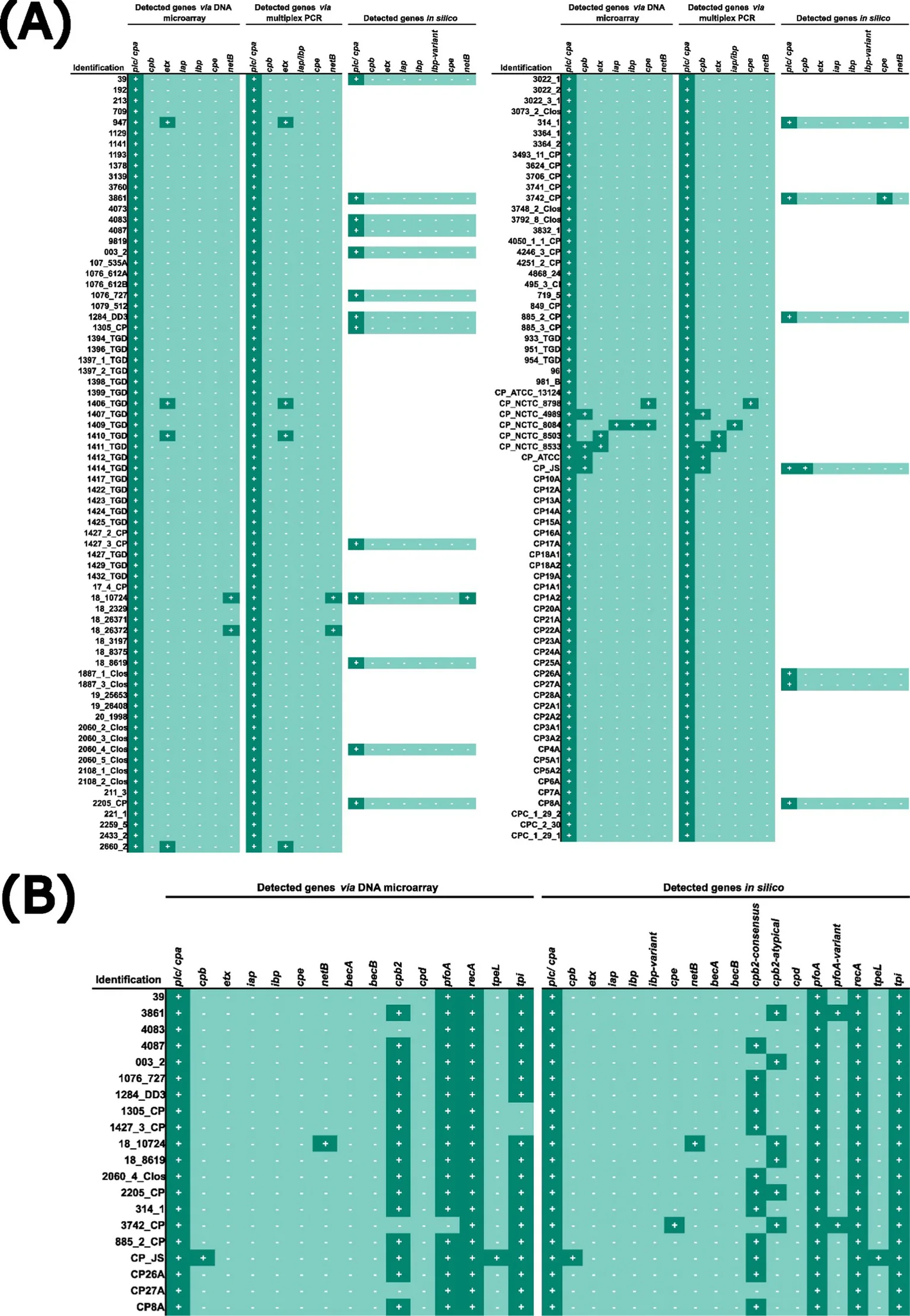
**(A)** Detection of typing toxin genes using DNA microarray, multiplex PCR, and *in silico* analysis. **(B)** Detection of *C. perfringens*-associated genes using DNA microarray and *in silico* methods. Presence (dark turquoise, “+”) and absence (light turquoise, “-“) of genes are indicated.

### 3.2 DNA microarray

The DNA microarray yielded positive signals for *plc/ cpa* (*n* = 143), *cpb* (*n* = 4), *etx* (*n* = 6), *iap* (*n* = 1), *ibp* (*n* = 1), *cpe* (*n* = 2), *netB* (*n* = 2), *cpb2* (*n* = 104), *cpd* (*n* = 3), *pfoA* (*n* = 136), *recA* (*n* = 143), *tpeL* (*n* = 4) and *tpi* (*n* = 132). No signals were detected for *becA* and *becB* in the tested strains (Figure 1). The DNA Microarray raw read data are available in Supplementary Table 3.

### 3.3 Multi locus sequence typing and goeBURST analysis

A total of 124 MLST analyses were performed (conventional *n* = 80, *in silico n* = 44), and the results are shown in Table 2. We were able to assign 77 different STs, 42 of which were novel. ST39 was the most abundant ST, totaling 30 strains. This was followed by ST850 with 5 strains, ST583 and ST856 with 4 strains each, ST820 and ST828 with 3 strains each and ST102, ST821, ST855 and ST859 with 2 strains each. Six different CCs were assigned. CC39 being the most prevalent, containing 15 different STs and 55 strains, followed by CC1025 with 3 STs and 5 Strains, CC583 with 2 STs and 5 strains, CC21 and CC80 with 3 STs and 3 strains each and CC1030 with 2 STs and 3 strains. The visualized goeBURST analysis can be seen in Figure 2.

**Table 2:** Multi locus sequence typing (MLST) results.

ID	CC	ST	*colA*	*groEL*	*sodA*	*plc/ cpa*	*gyrB*	*sigK*	*pgk*	*nadA*	Novel ST
18_10724	21	21	3	1	3	4	3	2	1	1	
18_26371		867	3	3	3	4	3	2	1	1	x
CP25A		1,028	228	1	3	4	3	2	1	1	x
96, 709, 1,129, 3,760, 4,087, 9,819, 107_535_A, 1076_612B, 1284_DD3, 1887_1_Clost, 1887_3_Clos, 2060_2_Clos, 2060_3_Clos, 2060_4_Clos, 2060_5_Clost, 2108_1_Clost, 2433_2, 3493_11_CP, 3706_CP, 495_3_Cl, 885_3, CP_JS, CP15A, CP19A, CP22A, CP26A, CPC_1_29_1, CPC_1_29_2, CPC_2_30, JXJA17_ncbi	39	39	33	4	40	39	7	4	3	19	
221_1, 314_1, CP10A		820	33	4	40	39	6	4	3	141	x
1305_CP		827	204	4	40	39	7	4	3	19	x
1076_612A, 1076_727, 1079_512		828	33	4	187	39	7	4	3	19	x
1427_3_CP		829	33	4	188	39	7	4	3	19	x
885_2_CP		830	33	4	40	39	7	149	3	19	x
1,141, 2108_2_Clost, CP12A, CP17A, CP6A		850	33	4	40	39	7	4	3	141	x
719_5		852	33	4	187	39	7	4	3	141	x
4246_3_CP		854	204	4	40	39	117	4	3	19	x
1427_2_CP, 3792_8_Clos, 4050_1_1_CP, CP21A		856	33	4	190	39	7	4	3	19	x
981_B		1,024	33	4	40	39	126	4	3	19	x
CP14A		1,026	33	4	40	39	113	4	3	19	x
CP23A		1,027	33	4	210	39	7	4	3	19	x
CP28A		1,029	33	4	40	17	7	4	3	141	x
CP7A		1,031	33	4	40	39	7	88	3	19	x
JP838_ncbi	80	79	41	55	44	65	23	26	21	40	
JFP914_ncbi		80	41	54	44	65	23	26	21	40	
JFP829_ncbi		718	41	54	44	65	23	105	21	40	
18_26372	583	122	52	1	3	51	3	4	1	1	
18_8619, 3022_1, 3022_2, 3022_3_1		583	4	1	3	51	3	4	1	1	
CP24A, CP8A	1,025	821	33	4	40	17	6	4	3	19	x
4073_CP, CP20A		855	33	4	40	17	117	88	3	19	x
CP13A		1,025	33	4	40	17	117	4	3	19	x
18_3197, CP15_ncbi	1,030	102	44	17	12	17	12	13	10	15	
CP4A		1,030	44	17	12	39	12	13	10	15	x
ATCC_13124	n.a.	1	1	1	1	1	1	1	1	1	
3742_CP		2	34	31	36	36	26	25	17	34	
NCTC8081_ncbi		31	21	12	16	20	14	15	13	18	
NCTC_8798		33	23	21	18	20	16	17	14	20	
19_25653		43	6	3	5	3	2	5	4	24	
MJR7757A_ncbi		74	62	15	57	66	10	21	9	57	
FORC_025_ncbi		77	4	34	1	6	4	4	3	1	
JP55_ncbi		78	4	19	3	4	3	5	20	1	
NCTC8679_ncbi		138	23	12	24	55	18	17	14	18	
NCTC10240_ncbi		140	55	43	41	56	35	32	15	45	
tumat_ncbi		146	63	46	60	67	38	40	8	59	
JS2202_ncbi		167	64	4	54	63	2	27	22	41	
Type_D_ncbi		169	59	52	64	64	40	39	9	1	
NCTC_8503		172	54	19	65	22	2	18	1	53	
NCTC8678_ncbi		173	23	12	24	20	16	17	14	54	
Q135.2_ncbi		177	61	44	56	38	37	26	26	62	
003_2		179	4	19	1	4	3	2	1	1	
CP_09_ncbi		255	36	33	38	69	55	25	18	36	
CP_33_ncbi		288	87	102	97	121	61	49	11	90	
20_1998		323	4	1	5	1	3	2	1	13	
FDAARGOS_903_ncbi		535	2	3	1	10	3	2	1	114	
2259_5		594	71	63	1	3	45	45	48	81	
NCTC10239_ncbi		595	23	26	24	20	16	17	14	20	
3741_CP		696	180	152	168	200	106	103	8	169	
19_26408		706	129	5	1	1	8	19	4	1	
CBA7123_ncbi		729	45	35	58	53	29	106	23	58	
3,861		772	83	168	52	210	55	133	18	168	
947		822	7	48	4	8	32	6	5	5	x
2205_CP		823	33	4	40	215	113	4	3	183	x
4,083		824	190	20	73	214	1	135	12	182	x
CP27A		825	148	173	4	216	2	136	1	184	x
39		826	162	99	1	54	11	4	8	62	x
192		848	25	16	191	9	43	4	8	11	x
213		849	164	172	193	213	50	53	86	181	x
1,193		851	88	95	89	103	25	12	4	152	x
4251_2_CP		853	33	135	40	21	7	4	3	19	x
3832_1		857	3	58	87	3	77	80	4	13	x
3748_2_Clos		858	188	14	48	10	2	151	81	198	x
3364_1, 3364_2		859	25	4	46	46	2	27	22	199	x
3,139		861	10	99	1	62	11	27	8	61	x
3073_2_Clost		862	33	181	40	233	7	4	3	19	x
2660_2		863	205	20	5	232	1	84	2	201	x
211_3		864	206	10	6	11	2	8	2	10	x
18_8375		866	114	113	189	230	64	101	8	202	x
18_2329		868	153	149	86	11	90	7	2	13	x
17_4_CP		869	207	58	1	3	2	2	1	13	x
1,378		871	3	94	192	231	118	150	8	200	x
849_CP		1,033	230	4	211	39	6	4	96	19	x
CP16A		1,038	12	80	1	264	7	56	97	217	x

The table presents assigned clonal complexes (CCs), sequence types (STs) and allelic profiles. Novel STs identified in this study are indicated by “x.” ID, identification; CC, clonal complex; ST, sequence type.

**Figure 2:**
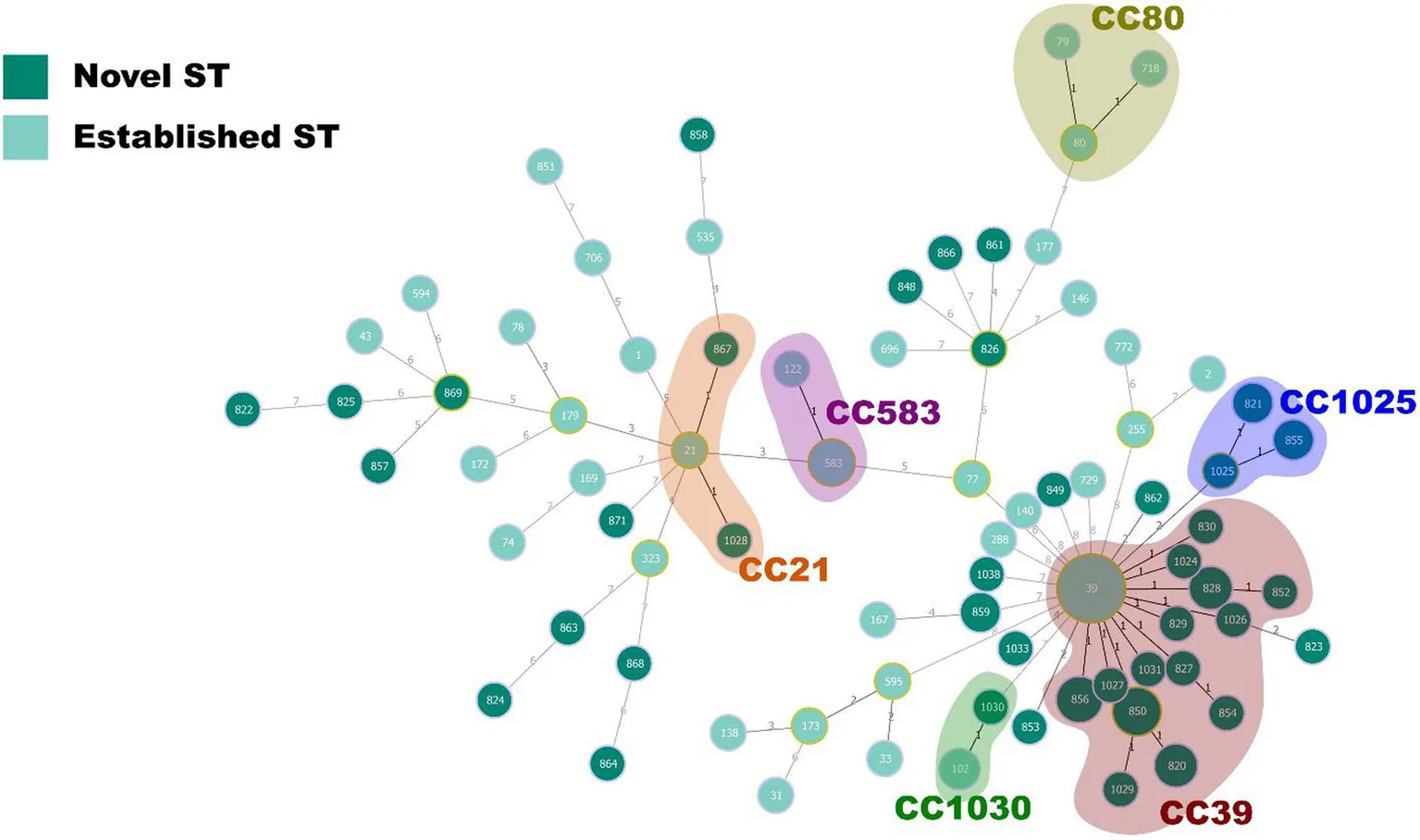
goeBURST analysis of Multi Locus Sequence Typing (MLST) results. Light turquoise circles represent established sequence types (STs), while dark turquoise circles indicate novel STs identified in this study. Numbers within the circles correspond to the assigned ST. STs grouped by background shading belong to the same clonal complex (CC), defined by a maximum of one allelic difference. CCs are named after the predicted founder (*e.g.*, the most central ST). Numbers on the lines connecting STs indicate the number of allelic differences. ST, Sequence Type; CC, Clonal Complex.

### 3.4 Maximum likelihood tree

Of the 124 analyzed isolates 6 strains were assigned to phylogroup I, 7 to phylogroup II, 101 strains to phylogroup III, 3 to phylogroup IV and 7 to phylogroup V. The generated maximum likelihood tree with further information can be seen in Figure 3.

**Figure 3:**
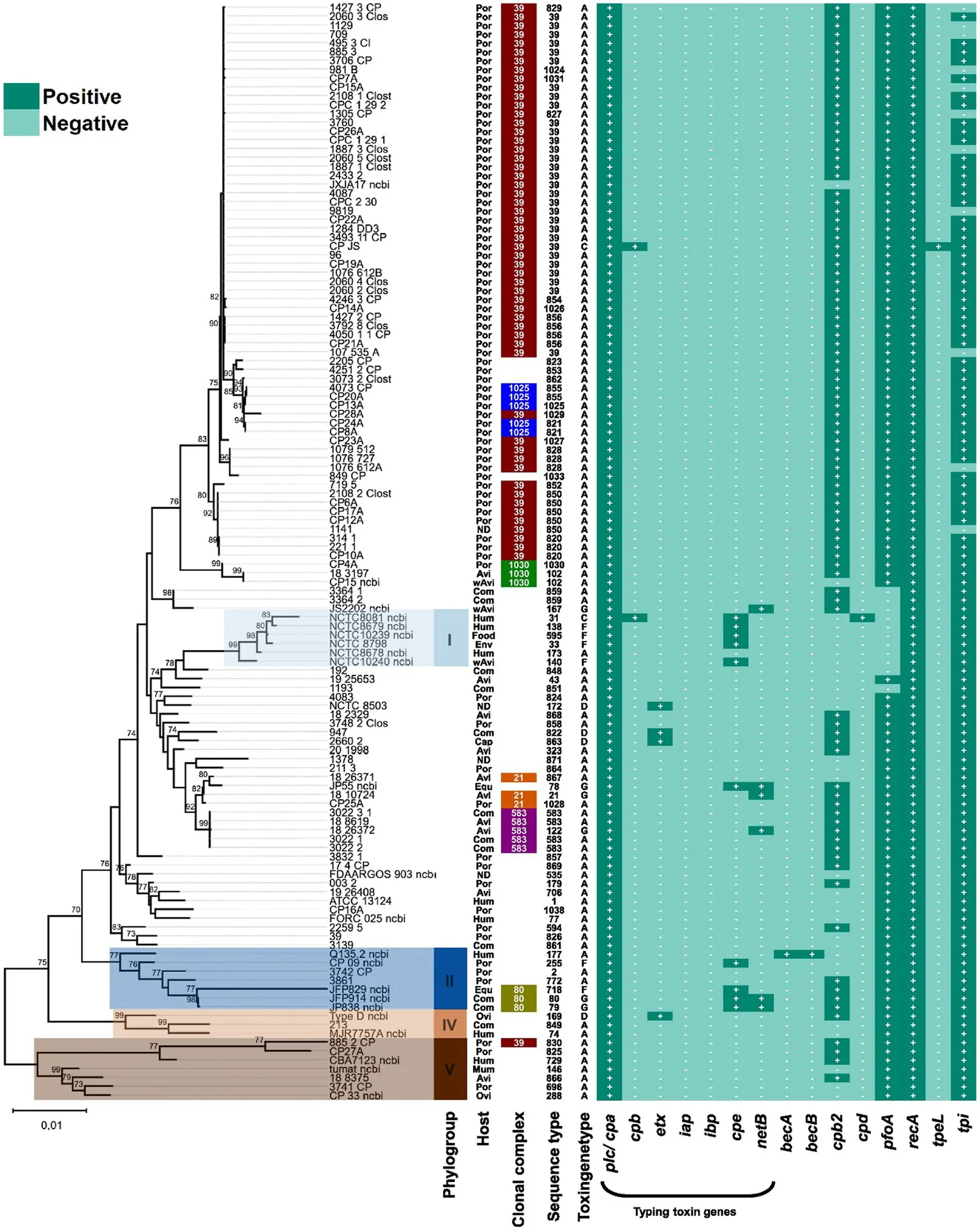
Maximum likelihood tree based on concatenated Multi Locus Sequence Typing (MLST) allele sequences. The tree was constructed using 1,000 bootstrap replicates; bootstrap values above 70% are displayed at the nodes. Strains are color-coded by phylogroup: Light blue (Phylogroup I), dark blue (II), light brown (IV), dark brown (V), and uncolored (III). Presence (dark turquoise, “+”) and absence (light turquoise, “-”) of genes are indicated. Phylogroups were assigned according to Abdel-Glil et al. (2021); sequence types according to the MLST scheme of Xiao et al. (2012); and toxin gene typing was performed according to Rood et al. (2018). Env, environment; Ovi, ovine; Por, porcine; Mum, mummy; Hum, human; Can, canine; Food, food associated; Equ, equidae; Avi, avian; Cap, caprine, wAvi, wild avian; ND, not defined.

### 3.5 *In silico* analysis of whole genome sequences

#### 3.5.1 Antimicrobial resistance associated genes

Antimicrobial resistance genes were present in 58.7% (*n* = 27) of tested genomes. Regarding the genomes of clinically associated strains generated in this study, 85% (*n* = 17) were positive for at least one AMR gene. In 23.9% (*n* = 11) of genomes at least one AMR gene and in 34.8% (*n* = 16) at least two different AMR genes were detected. Isolate 885_2_*C. perfringens* harbored 9 different AMR genes against the antimicrobial (AM) classes of aminoglycosides, pleuromutilin, lincosamides, macrolides, streptogramins and tetracycline. The most abundant AMR genes identified were against tetracyclines (*tet*(44) *n* = 12, *tet*A(P) *n* = 23, *tet*B(P) *n* = 6). There were 5 different genes present (*aac(6′)-Ie/aph(2″)-Ia n* = 1, *aad9 n* = 1, *aadE n* = 1, *ant(6)-Ib n* = 12 and *aph(2″)-If n* = 2), which are associated with resistances against aminoglycosides. Against the AM classes pleuromutilin, lincosamide, macrolide and streptogramin four different genes (*erm*(B) *n* = 1*, erm*(Q) *n* = 3*, lnu*(B) *n* = 1 and *lsa*(E) *n* = 1) were detected (Figure 4; Supplementary Table 4).

**Figure 4:**
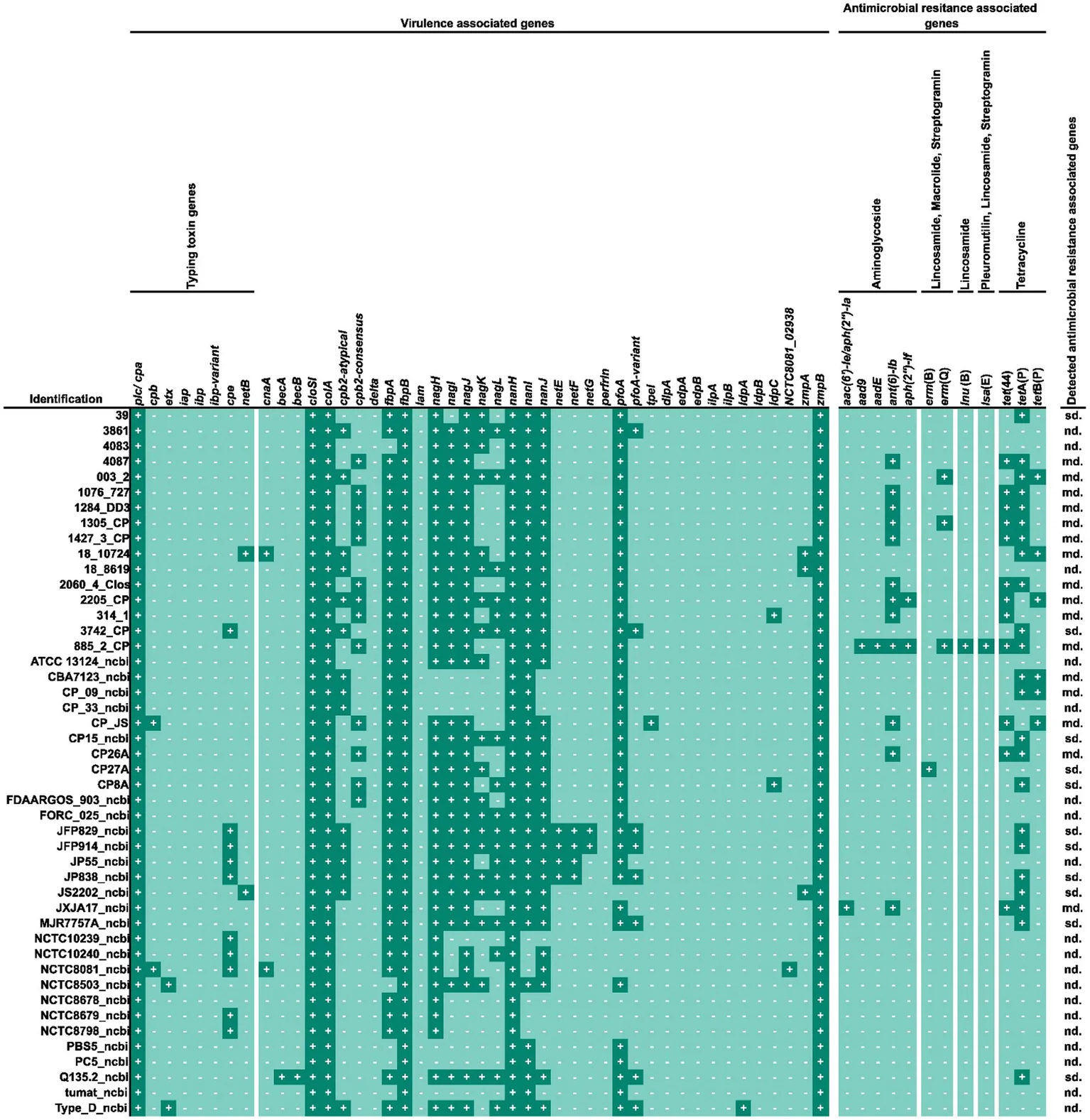
Detected potential virulence and antimicrobial resistance associated genes *in silico*. Presence (dark turquoise, “+”) and absence (light turquoise, “-“) of genes are indicated. Nd., non detected antimicrobial resistance associated (AMR) genes; Sd., single detected AMR gene; Md., multiple detected AMR genes.

#### 3.5.2 Virulence associated genes

In all 46 *in silico* screened *C. perfringens* strains the genes *plc/ cpa, cloSI, colA, fbpB, nanH* and *zmpB* were detected. None of the screened isolates harbored the genes *iap, ibp, ibp-variant, delta, lam, perfrin, dlpA, edpA, edpB, ilpA, ilpB* and *ldpB*. The genes *fbpA, nagH, nanI, pfoA, nagJ, nanJ, nagI, nagK, nagL, cpb2-atypical, cpb2-consensus, cpe, pfoA-variant, netE, netF, zmpA, cpb, etx, netB, cnaA, netG, ldpC, becA, becB, tpel, ldpA* and *NCTC8081_02938* were detected at least once (Figure 4; Supplementary Table 4).

No biocide resistance genes were detected *in silico* by the method applied.

## 4 Discussion

Comprehensive genomic characterization and analysis of *C. perfringens* strains in Europe remains scarce in the literature. However, *C. perfringens* and its toxins are regularly one of the top 10 causes of very large food-borne outbreaks and one of the top three agents in vulnerable settings (i.e., healthcare and residential facilities, school and workplace canteen) in the European Union. Besides inadequate chilling and storage time/ temperature abuse outbreaks were associated with meat and its products, mixed foods and vegetables/ juices products (European Food Safety Authority (EFSA) and European Centre for Disease Prevention and Control (ECDC), 2025). Songer (2010) discussed that Clostridia spp.– including *C. perfringens* – are not considered to be potent zoonotic pathogens. Diseases in human suffering from *C. perfringens* infections mostly occur due to contaminated and improperly handled food. The toxin CPE being responsible for the usually self-limiting intestinal disease. Livestock are infrequently infected by CPE producing *C. perfringens*. However, more serious human infections with CPB producing strains are possible but are rarely described. A recent study showed a detection rate of *C. perfringens* along the slaughter process of 19.7–25.5%, therefore posing a potential risk of product contamination with the bacterium, but *cpe* was not detected in the strains isolated (Tran et al., 2026). The zoonotic potential of *C. perfringens* was discussed by Hamza et al. (2018) as they isolated related strains in diarrhetic humans and heathy livestock according to *cpa* typing. We identified a knowledge gap, as in depth epidemiological analysis of *C. perfringens* is missing. To the best of the authors’ knowledge, this is the first in depth study of this kind conducted in Austria. Sipos et al. (2003) genotyped *C. perfringens* isolated from Austrian ruminants and swine according to the presence of five toxin genes and Boattini et al. (2025) performed a retrospective analysis of most common detected anaerobe bacteria in European hospitals, where an Austrian clinic took part. Phenotypic antimicrobial resistance testing followed. However, no further in depth epidemiological analysis was performed. This is further supported by the absence of publicly available Austrian *C. perfringens* isolates of human, animal or environmental origin in the PubMLST database at the onset of the study. To elucidate this blind spot, we performed conventional and *in silico* MLST on non-repetitive clinical *C. perfringens* isolates derived from gut-associated samples of Austrian animals.

We identified 42 novel STs and assigned six clonal complexes. On PubMLST no predefined CCs are currently published for *C. perfringens*. We were able to identify porcine associated CCs and STs which we considered as related (i.e., up to two alleles difference). All isolates of CC39, CC1025, ST823, ST853 and ST862 were derived from porcine associated samples, except ID 1141 of CC39 of which the source is not known. In the maximum likelihood tree these strains cluster together in a branch (bootstrap value of 83) and can all be grouped to phylogroup III, except ID 885_2_*C. perfringens* which is categorized in phylogroup V. Furthermore, ST1025 and ST853, and ST862 can be considered as related to CC39, as they only differ in two alleles. ST853 and ST862 differ in *groEL* and *plc* and ST1025 differs in *plc* and *gyrB*. On PubMLST there are two entries with an exact match for ST39 (i.e., JGS1495 and NobL1JXJA17), two with up to one allelic difference (i.e., CP-05 and 6076) and a single entry with up to two allelic differences (i.e., 1,860,308). The source of three strains is unknown and of two it is only known that they were isolated from animals. Therefore, our data indicates, that CC39 and its close relatives, are porcine associated and adapted to the porcine host. Zekun et al. (2026) most recently reported MLST characterization of *C. perfringens* strains isolated from the Xinjiang region in China of porcine specimens. When compared with our MLST analysis, 17 described and 1 novel STs (i.e., ST893) showed a maximum of two alleles difference to ST39 and therefore can either be assigned to CC39 or considered as related as described above, supporting our claim of a porcine associated CC (Supplementary Figure 1). Furthermore, Song et al. (2024) reported that 7 *C. perfringens* strains isolated from swan geese (*Anser cygnoides*) clustered exclusively in CC1 whereas *C. perfringens* strains isolated from other distinct wild avian species did not cluster in a specific CC. The authors discussed an adaptation to the host. The ability of *C. perfringens* to adapt to niches was discussed by Abdel-Glil et al. (2021) as well. Strains belonging to phylogroup I – mostly associated with food poisonings in humans – have an overall smaller genome size, a limited number of insertion sequences which are more common and the *cpe* gene is often encoded on the chromosome rather than on a plasmid. Therefore, it was theorized by the authors, that the strains in phylogroup I are adapted to replicate more readily on food matrices (Abdel-Glil et al., 2021).

*C. perfringens* is a pathobiont commonly present in the normal intestinal microbiota of both animals and humans. Therefore, isolation of the bacterium alone from gut-associated clinical samples does not necessarily confirm its involvement in enteric disease (Kiu and Hall, 2018; Mehdizadeh Gohari et al., 2020) and should be complemented by additional diagnostic investigations. *C. perfringens* is routinely classified using a toxin gene based typing scheme, in which isolates are assigned to toxinotypes (Type A–G) based on the presence of six typing toxin genes (Rood et al., 2018). Those toxin-genes used for classical typing do not cover the whole virulence repertoire of the pathogen, as more than 20 assumed virulence associated hydrolytic enzymes and extracellular toxins are currently described for *C. perfringens* (Revitt-Mills et al., 2015; Camargo et al., 2024). To account for the fulfillment of molecular Koch’s postulates Rood et al. (2018) updated the toxin gene typing scheme to include *C. perfringens* Type F (responsible for human food poisoning, non-food poisoning and antibiotic associated diarrhea; solely *plc* and *cpe* positive) and *C. perfringens* Type G (associated with necrotic enteritis in fowl; solely *plc* and *netB* positive). Furthermore, Mehdizadeh Gohari et al. (2020) summarized evidence that canine acute hemorrhagic diarrhea syndrome and foal necrotizing enteritis can be associated with *C. perfringens* Type A strains harboring *netF*, a toxin-gene-combination currently not covered by the updated and commonly used typing scheme (Rood et al., 2018). It is therefore possible that further novel toxin gene types will be described in the future, provided that they correlate with clinical cases, epidemiological data and fulfillment of molecular Koch’s postulates. A diagnostic tool is therefore needed to examine clinical *C. perfringens* strains for a broader panel of potential virulence associated genes. Hence we tested a novel DNA microarray including 15 *C. perfringens* associated genes to perform expanded, rapid and simultaneous *C. perfringens* genotyping in a single reaction – breaching the gap between routine diagnostics and comprehensive virulence gene typing.

Conventional single PCR has comparable lower throughput and the workload of laboratory staff increases drastically for each individual target gene (Elnifro et al., 2000). The sensitivity and specificity of conventional multiplex PCR sharply decreases at a certain point, limiting the number of target genes (Shafiei-Jandaghi et al., 2023). Therefore, a broad-range DNA microarray for the detection of virulence genes was developed as part of this study. DNA microarrays are powerful tools for the rapid, simultaneous, and resource-efficient detection of genes in bacteria. DNA microarrays have been successfully used to detect 144 different gene targets in *Staphylococcus aureus* (Monecke et al., 2007) and 67 different genes in *Escherichia coli* (Bernreiter-Hofer et al., 2021). In addition to detecting virulence genes, microarrays can also be used to detect genes associated with antibiotic resistance (Perreten et al., 2005).

We compared the results of the DNA microarray with multiplex PCR and *in silico* analysis of selected WGS strains generated in this study to detect the six typing toxin genes. Overall, the different methods were highly comparable and yielded similar results. One exception was isolate ID 3742_CP, where *cpe* was detectable *in silico* but could not be detected with the DNA microarray and multiplex PCR. Furthermore, *cpe* could not be detected with multiplex PCR in ID CP_NCTC_8084, whereas the DNA microarray detected the gene.

All genes present on the DNA microarray were detected at least once, with the exception of *becA* and *becB.* The other methods used did not detect those genes either in the tested strains. Overall, the prevalence of those two genes is considered to be rather low, for human *C. perfringens* isolates a detection rate of 1/798, 1/195, 1/132 and 1/129 has been reported, for companion animals 1/59, for bovine 1/50 and for swine 1/62 (Yonogi et al., 2016; Kiu et al., 2019; Matsuda et al., 2019; Fang et al., 2025; Zekun et al., 2026). Unfortunately, we were unable to obtain a strain with these genes to verify whether the DNA microarray would have detected them.

Furthermore, we detected *cpe* and *netB* simultaneously in three publicly available genomes (i.e., ID JP55, ID JP838 and ID JFP914). Strain JP55 was isolated of an equine host from Canada in 2007. The publicly available information does not state a disease background. Isolate JP838 was cultivated form a dog with hemorrhagic gastroenteritis from the USA in 2009. Strain JFP914 was isolated of a dog suffering from canine acute hemorrhagic diarrhea syndrome from Switzerland in 2017. According to the toxin gene typing scheme currently in use (Rood et al., 2018), these three strains could not be assigned to a toxin-gene-type. Most recently Mada et al. (2025) described a *C. perfringens* strain, isolated from a suddenly diseased Holstein cow in Japan, which was positive for *cpe* and *netB*. Therefore, we can support their claim, that the currently used toxin gene typing scheme for type G should be adapted so that *cpe* is tested either positive or negative, as is the case for type C, D, and E.

Anaerobes in general are intrinsically resistant to aminoglycosides, which can be explained by the oxygen dependent transport of the substance into the bacterial cytoplasm. In addition, natural resistance to polymyxins and some fluoroquinolones has been described in anaerobic bacteria. The genus *Clostridium* spp. is additionally intrinsically resistant to trimethoprim due to a permeability barrier and the insensitivity of the enzyme dihydrofolate reductase to the substance (Schwarz et al., 2018). Although *C. perfringens* is intrinsically resistant to aminoglycosides (Schwarz et al., 2018), aminoglycoside-associated resistance genes were the second most frequently detected AMR genes in our dataset, occurring in 26.1% of strains (12/46). All 12 strains harbored at least two different AMR genes against aminoglycosides and were positive for *ant(6)-Ib*. The other detected genes (i.e., *aac(6′)-Ie/aph(2″)-Ia, aad9, aadE* and *aph(2″)-If*) were scarcer. The *ant(6)-Ib* gene, encoding for an aminoglycoside modifying enzyme, is located on a transferable pathogenicity island that also contains *tet*(44) (Abril et al., 2010). Our results are consistent with those of Abril et al. (2010) as all strains were positive for *tet*(44) and *ant(6)-Ib,* and none of those genes were detected alone.

A total of 27 out of 46 (58.7%) *C. perfringens* WGS tested positive for at least one AMR gene. Assessing only the clinical strains generated in this study, 17 out of 20 (85.0%) strains tested positive for at least one AMR gene. These results are consistent with the findings of Camargo et al. (2022), who were able to detected AMR genes *in silico* in 72.8% of 372 examined strains. In contrast Zhong et al. (2023) reported at least one AMR in 97.1% of 178 *C. perfringens* strains tested from different regions in China. This could support the claim, that AMR gene distribution is dependent on the region and local habits in AB usage (Schwarz et al., 2018).

We detected the highest number of AMR genes against tetracyclines. Overall, 26 of 46 strains (56.5%) carried *tet*(44), *tet*A(P) and/ or *tet*B(P). The genes *tet*(44) and *tet*B(P) confer resistance through ribosomal protection, whereas *tet*A(P) encodes an efflux pump (Chopra and Roberts, 2001; Abril et al., 2010). Furthermore, *tet*A(P) and *tet*B(P) are encoded on an operon that is transcribed from a single promoter (Johanesen et al., 2001). Our findings are comparable to those of Anju et al. (2021), who identified tetracycline resistance genes in 41.3% (31/75) of *C. perfringens* isolates by PCR. They detected *tetA*(P) in 13 isolates, *tet*B(P) in 2, and both genes in 16 isolates, while *tet*(M), *tet*(L), and *tet*(K) were not present. However, *tet*(44) was not included in their screening panel. Similarly, Kiu et al. (2017) reported *tet*A(P) in 75% (42/56) of isolates and the combination *tet*A(P) + *tet*B(P) in 23%. In our dataset, *tet*A(P) + *tet*B(P) occurred together in four strains, *tet*(44) + *tet*A(P) was detected in nine strains and *tet*(44) + *tet*B(P) in two strains. We did not detect *tet*B(P) alone, but only in combination with *tet*A(P) or *tet*(44). Zhong et al. (2023) reported a much higher overall prevalence of tetracycline resistance genes (97.2%; 173/178 isolates), with strains harboring *tet*A(P), *tet*B(P), *tet*(44), or combinations thereof. The most common profile was *tet*A(P) + *tet*B(P) (71.4%), followed by *tet*A(P) + *tet*(44) (14.6%). Furthermore, they detected *tet*A(P) + *tet*B(P) + *tet*(44) in 6.2% of isolates and *tet*B(P) alone in 1.7%, whereas *tet*(44) alone was not observed.

Isolate 885_2_*C. perfringens* of our study was exceptional, not only as it harbored 9 different AMR genes simultaneously (i.e., *aad9, aadE, ant(6)-Ib, aph(2″)-If, erm*(Q), *lnu*(B), *lsa*(E), *tet*(44) and *tetA*(P)). Even more interestingly the strain was positive for the gene *lsa*(E), an ABC transporter protein conferring resistance to pleuromutilin, lincosamide and streptogramin (Wendlandt et al., 2013). Up to the authors knowledge this is the first description of *lsa*(E) for *C. perfringens* (National Center for Biotechnology Information, 2016; Schwarz et al., 2018). The presence of more than one AMR gene in *C. perfringens* is well described (Adams et al., 2018; Yadav et al., 2022) and recently a *C. perfringens* strain harboring up to 19 different AMR genes was published (AlJindan et al., 2023).

In our WGS analysis, no AMR genes associated with beta-lactam antibiotics were detected, although beta-lactams are considered the first-line drug class for treating *C. perfringens*-associated diseases in animals (Danish Veterinary and Food Administration, 2018; Uzal and Songer, 2019; Cutler et al., 2020; Andina-Pfister et al., 2022). Up to the authors knowledge, no data is available on treatment approaches for *C. perfringens* related diseases in animals in Austrian extramural care. However, in a large scale humane medicine focused multicenter retrospective observational study in Europe phenotypic susceptibility in *C. perfringens* for beta-lactams was over 90% (Boattini et al., 2025).

We performed epidemiological comparison according to MLST results. Cross verification with virulence and AMR gene profiles was not performed. Geier et al. (2021) showed that some pore forming toxins of *C. perfringens* appears to be host associated.

We used exclusively an *in silico* approach for the detection of AMR genes, which is a limitation of this study. Phenotypic resistance testing *in vitro* was not in scope of this publication. Further studies should be conducted on the correlation of genotypic and phenotypic antibiotic resistance testing to clarify whether the *in silico* prediction approach works as well for *C. perfringens* as described for other intestinal bacteria (Feldgarden et al., 2019).

Furthermore, no analysis was conducted to determine whether virulence and AMR genes were located on the plasmid or on the chromosome of the strains. As virulence and AMR genes can be encoded on plasmids and/ or chromosomes in *C. perfringens*. Plasmid encoded genes are often associated with mobile elements and facilitate gene transfer between *C. perfringens*, aiding the pathogens ability of virulence plasticity and adaptability (Li et al., 2013).

In addition, we only described *in silico* detected potential virulence associated genes. No *in vitro* and/ or *in vivo* testing was performed to verify, if the identified genes actually got transcribed and bacteria produced the corresponding extracellular toxin and/ or hydrolytic enzyme.

Overall, publicly available information on the epidemiology of *C. perfringens* remains limited, particularly in Europe. On PubMLST, only around 1,700 isolates and approximately 700 genome datasets have been deposited. In contrast, for *Staphylococcus aureus* approximately 48,000 isolates^3^ and for *Escherichia* spp. around 421,000 isolates^4^ are deposited in public databases. Our strain collection is limited to *C. perfringens* of Austrian animals, lacking human and environmental isolates. Therefore, efforts should be made to ensure more information on *C. perfringens* in humans, different animals and environmental niches is available and made accessible in publicly database to facilitate future outbreak investigations, tracking spillover events and conducting general epidemiological research on *C. perfringens*.

The tested DNA microarray does not depict the current plethora of described potential virulence associated genes for *C. perfringens.* We plan to adapt the DNA microarray to include the assumed virulence associated genes screened *in silico* in this study (Figure 4). The platform is capable of such an adaptation. In addition, we were able to demonstrate the benefit of using a microarray for routine diagnostics, as a larger panel of genes could be investigated simultaneously and deeper insights into the carriage of virulence associated gene of *C. perfringens* isolates could be gained. From a sustainability perspective, our microarray based assay saved time and labor of laboratory staff.

In summary, the Austrian isolate collection showed substantial genetic diversity, including 42 novel sequence types and six clonal complexes, underscoring the limited prior epidemiological characterization of this pathogen in Austria. In addition, the predominance of CC39 among porcine isolates suggests that this lineage may represent a porcine-associated clonal complex, a hypothesis that warrants further investigation *via* comprehensive typing techniques (e.g., MLST, cgMLST) and validating the host specifity of virulence genes in larger strain collections, across additional host species and in different geographical locations. Our findings indicate that the custom DNA microarray is a practical and efficient screening tool for expanded *C. perfringens* genotyping, with performance comparable to multiplex PCR and *in silico* WGS based analysis.

## Data Availability

The datasets presented in this study can be found in online repositories. The names of the repository/repositories and accession number(s) can be found at: https://www.ncbi.nlm.nih.gov/, PRJNA1495884; https://www.ebi.ac.uk/ena, PRJEB121207.

## Footnotes

1 https://pubmlst.org/organisms/clostridium-perfringens; accessed at 19.07.2026.

2
www.pubmlst.org

3 https://pubmlst.org/, access date 19.07.2026.

4 https://enterobase.warwick.ac.uk/, access date 14.01.2026.

## Supplementary material

SUPPLEMENTARY FIGURE 1 Comparison of detected Sequence Types (ST) via Multi Locus Sequence Typing (MLST) from Zekun et al. (2026) and this publication. The digit in the circle refers to the ST, and the size indicates the number of STs detected. The number beside the connecting line shows the number of allelic differences between ST’s. Panel **A** shows a comparison of the national origin of the detected STs. Color indicates the country of detection. In panel **B**, the hosts of the respective STs are compared. Color indicates the host species.

SUPPLEMENTARY TABLE 1 Isolates and genomes used in this study.

SUPPLEMENTARY TABLE 2 DNA quality and quantity of used isolates.

SUPPLEMENTARY TABLE 3 DNA Microarray raw read data.

SUPPLEMENTARY TABLE 4 *In silico* detected virulence and AMR genes.
